# Progress and Challenges in Canada’s Path Toward the Elimination of Cervical Cancer

**DOI:** 10.3390/curroncol31100435

**Published:** 2024-09-29

**Authors:** Samara Perez

**Affiliations:** 1 Department of Oncology, Faculty of Medicine and Health Sciences, McGill University, Montréal, QC H4A 3T2, Canada; samara.perez@mcgill.ca; 2 Research Institute of McGill University Health Centre, Montréal, QC H4A 3J1, Canada; 3 Psychosocial Oncology Program, Cedars Cancer Centre, Division of Supportive & Palliative Care, McGill University Health Centre, Montréal, QC H4A 3J1, Canada

**Keywords:** cervical cancer elimination, human papillomavirus, HPV vaccination, cervical screening, Canada, HPV DNA testing, vaccine coverage, public health strategy, cancer prevention, health disparities

## Abstract

Cervical cancer is almost entirely preventable and treatable when detected early, making its elimination within reach for Canada and the world. However, cervical cancer is now the fastest-increasing cancer (+3.7% per year since 2015) in Canada as of 2023, marking the first significant increase in cervical cancer incidence since 1984. The human papillomavirus (HPV) vaccine and cervical screening are key preventive measures, with targets set by the WHO and the Canadian Partnership Against Cancer (CPAC) to eliminate cervical cancer in Canada by 2030 and 2040, respectively. These targets include increasing HPV vaccination rates, implementing primary HPV screening, and improving follow-up for abnormal HPV+ results. However, Canada’s progress has been impeded by significant challenges. As of the most recent data, HPV vaccine coverage rates in Canada range from 47% to 81%, with an estimated national HPV vaccination completion rate of 64% in Canada, far below the target of 90% by 2025 set by the CPAC. With the exception of British Columbia and Prince Edward Island, the adoption of HPV DNA testing as the primary screening method has been slow across the Canadian provinces and territories despite its superior sensitivity compared with traditional cytology. This article reviews the current state of HPV vaccination and screening in Canada, emphasizing the need for coordinated efforts, transparency, and resource sharing to overcome barriers. Key recommendations include the dissemination of accessible educational materials, partnerships, and collaboration, including nationwide task forces and roundtables, and the implementation of standardized guidelines for HPV screening. Achieving cervical cancer elimination requires a united approach involving federal, provincial, and territorial health authorities, researchers, clinicians, NGOs, community groups, and patients’ voices working together to ensure consistent, effective, timely, and meaningful cervical cancer prevention strategies are used across the country.

## 1. Introduction

Cervical cancer is almost entirely preventable and treatable when detected early. As such, cervical cancer can be eliminated (defined as 4 or fewer cases per 100,000 annually) in Canada and worldwide. The human papillomavirus (HPV) vaccine and cervical screening are safe and effective primary and secondary prevention measures, respectively, for HPV-related diseases associated with HPV [1]. HPV can cause cervical, head and neck, vaginal, vulvar, anal, and penile cancers, as well as genital warts. In Canada, the Pap test (also known as the Pap smear) has been traditionally used for over 60 years as a primary method for the early detection of cervical cancer. All provinces except Québec have organized cervical screening programs. An explanation of the setup of these organized cervical screening programs (in 2021) can be found in [2]. However, there is now a significant shift toward using HPV DNA testing, which offers more sensitive and accurate detection of high-risk HPV infections, the primary cause of cervical cancer [2].

As healthcare in Canada is managed at the provincial and territorial levels, each of the 10 provinces and 3 territories has the autonomy to decide its cervical cancer prevention strategy. At a national level, guidelines and indicators can be found in the Canadian Task Force on Preventive Health Care Programs in Canada, prepared by the Screening Performance Indicators Working Group, the Cervical Cancer Prevention and Control Network, in 2009, with an update expected in 2025 [3]. There was an update from the Canadian Task Force on Preventive Health Care that provided recommendations on screening for cervical cancer in 2013, with proposed performance indicators included in Appendix 7 of their recommendations [4]. Both of these guidelines require updating. In 2021, a protocol was published with the goal of informing recommendations by the Canadian Task Force on Preventive Health Care for cervical cancer screening in primary care by systematically reviewing evidence on effectiveness, test accuracy, individuals’ values and preferences, and strategies to improve screening rates [2]; however, to the best of our knowledge, the resulting article has not yet been published. At the provincial level, each province has its own set of indicators, which vary greatly depending on the status of the implementation of HPV testing in that province [5,6] and, therefore, are less defined at this time as we are in a transition period to HPV DNA testing.

In response to the World Health Organization (WHO)’s goal to eliminate cervical cancer globally this century [7], the Canadian Partnership Against Cancer (CPAC), in collaboration with various national partners, developed the Action Plan to Eliminate Cervical Cancer in Canada, 2020–2030 [8]. This plan focused on three main priorities [8]:

(1) Increase HPV vaccination rates, namely, a target set that by 2025, 90% of 17-year-olds will be fully vaccinated with the HPV vaccine (in line with the National Advisory Committee on Immunization (NACI)’s call to action).

(2) Implement HPV primary screening, namely, a target set that by 2030, 90% of eligible individuals will have been screened with an HPV test; that 90% of eligible individuals will be up to date with cervical screening; and that no less than 80% of eligible individuals in any identifiable group will be up to date with cervical screening.

(3) Improve follow-up for abnormal results, namely, a target set that by 2030, 90% of all individuals with an abnormal screening result (HPV+ test) should have a clear plan of appropriate follow-up designed and communicated to them within three months of the test that generated the positive result; 90% of all individuals identified as being at elevated risk for significant cervical abnormalities will have a colposcopy in a timely manner; and no less than 90% of individuals in any identifiable group will receive follow-up.

These triple intervention targets are acting simultaneously on three key fronts. The plan also addresses specific inequities faced by First Nations, Inuit, and Métis in cervical screening [8].

At the time of writing this article, we are four months away from 2025 and approaching the halfway mark to 2030 (two of three CPAC targets [8]). This article will provide an update and overview of the first two priorities listed. Given that healthcare in Canada is managed at the provincial and territorial levels, and due to the separation between immunization and screening services, compiling data required extensive email correspondence and research. First, the peer-reviewed literature was extensively searched. Second, to the best of our capacities, the grey literature was also searched, which included contacting provincial/territorial health agencies, provincial cancer societies/agencies, colleagues, researchers, and clinicians across the country to retrieve the most up-to-date HPV coverage rates and assess the status of HPV primary screening implementation per province and territory. The rationale was to have all of this information up-to-date and synthesized in one article. The triumphs and ongoing hurdles with respect to cervical cancer elimination in Canada that remain will be discussed, along with key recommendations of where to direct our efforts to achieve cervical cancer elimination in Canada.

### HPV Vaccination in Canada

In Canada, publicly funded school-based HPV immunization programs were first implemented for females from 2007 to 2013, with males added from 2013 to 2017 [9,10]. HPV immunization is now offered to all children in all provinces and territories in school-based programs between Grades 4 and 7, depending on the province or territory [9,10,11]. Children can also receive the HPV vaccination in older grades, e.g., Grades 9 to 12. These are commonly referred to as catch-up programs. Depending on the province and territory, most individuals aged 18 to 26 can get the HPV vaccine for free, but it likely requires some effort since it is not part of the school programs [9,12]. There are also additional eligibility criteria for those who are immunocompromised, HIV-positive, men who have sex with men, and males in youth custody service centers, irrespective of age, who can receive the HPV vaccine within publicly funded programs, though this, again, depends on the province and territory they reside in [9,12]. Except for Québec, which uses Gardasil^®^9 for the first dose and Cervarix for the second dose, the rest of Canada uses the nonavalent Gardasil^®^9 in a two- or three-dose schedule [13].

As of 24 July 2024, the NACI has updated its recommendations for the HPV vaccine [14]. Individuals between the ages of 9 and 20 who are not immunocompromised should receive a single dose of the HPV vaccine. For those aged 21 to 26, two doses are recommended unless they have compromised immune systems. For individuals aged 27 and older, a two-dose schedule may be considered in consultation with an HCP. Furthermore, the HPV vaccine can be offered during pregnancy. Routine questioning about the last menstrual period and/or pregnancy is not required or recommended before offering the HPV vaccine. It is still unknown how this recent one-dose model will impact each provincial program.

**Figure 1:**
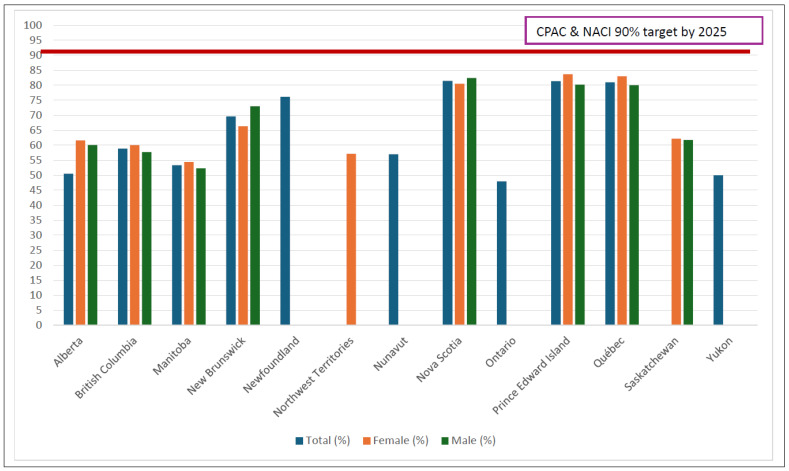
HPV vaccine completion rates by province.

Across all provinces, pre-pandemic rates for HPV vaccine completion in school-based programs ranged from 57 to 91% [2]. The COVID-19 pandemic greatly impacted HPV school-based immunization programs [15,16]. As an example, in New Brunswick, approximately 1600 HPV vaccines were administered from January 2021 to August 2021, compared with 5650 in the previous year [17]. Since current, up-to-date HPV rates are difficult to find in one place and not always up to date, we provide the most recent data on HPV vaccine coverage rates in Canada, as shown in Table 1 and Figure 1. In the post-pandemic COVID-19 era, there has been an important decline in school-based HPV completion rates, ranging from 47 to 81%. Therefore, Canada is at great risk of not achieving the CPAC target to have 90% of 17-year-olds fully vaccinated with the HPV vaccine by 2025. Thousands of youth remain vulnerable to the morbidity and mortality associated with HPV cancers. This is a clear crisis in the provision of HPV vaccination, which has not been adequately recognized or addressed adequately by vaccine providers. While some provinces have tried to mitigate this by administering more vaccines through public health teams, we are still seeing the consequences of low rates of HPV vaccination. This is seen despite Canada having COVID-19 immunization uptake at 85% for one dose and at 82% of the primary series completion % coverage [18], suggesting that the majority of Canadians are not anti-vaccination and/or vaccine-hesitant, but there has been a lack of clear, targeted messaging and campaigns to get Canadians vaccinated against HPV.

## 2. The Implementation of Shifting from Cytology to HPV Testing in Canada

It is an exciting time, as we have entered into a new era for cervical screening. The advancement of the HPV DNA test, a superior test that has greater sensitivity in detecting pre-cancerous lesions, allows for longer intervals between screenings [30,31]. The HPV test is the recommended test for primary cervical cancer screening by the leading national [32] (e.g., The Society of Obstetrics and Gynecologists of Canada) and international organizations (the WHO) [33,34,35,36] in women’s health and the gynecological and oncological spheres. HPV testing can be performed by a healthcare provider (HCP) or performed by oneself through self-sampling (either at home or at the clinic), a true “game changer” to overcome barriers among the under-screened population, which often includes those who identify as Indigenous or immigrants and those who live in remote and/or rural communities [37]. Self-sampling completely mitigates known barriers, including access to an HCP and/or access to a clinic (e.g., time and transportation), as well as fear and embarrassment that can be experienced with an HCP.

Initially, the Canadian newspapers buzzed, and some were quoted as saying, “There’s huge momentum happening in the country to make that switch” [38]. However, Canada’s transition has been extremely slow. Based on both the academic and grey literature, Table 2 provides the most recent up-to-date statuses of the provinces and territories in their shift to HPV DNA testing as the primary method for cervical cancer screening.

## 3. Recommendations

The CPAC’s Cervical Cancer Elimination strategy is part of a broader Canadian Strategy for cancer control encompassing eight key priorities [47]. Among these, priority one focuses on decreasing the risk of cancer by adopting proven practices, specifically increasing HPV vaccination rates and implementing HPV screening for cervical cancer. While the goals are well defined, we must ensure that we take actionable strategies to achieve and realize these goals.

Examining the current total coverage rates in Table 1, 6 out of 11 provinces/territories have HPV vaccine completion rates below 65%. We can estimate a 64% national HPV vaccination completion rate in Canada, which is far from the goal of 90% HPV vaccination coverage for Canadians by age 17 in 2025. Moreover, although HPV vaccination programs in Canada are school-based, the coverage rates remain lower than those observed in other countries with similar school-based vaccination strategies, indicating that additional efforts may be needed to improve uptake and achieve higher vaccination rates. This highlights the importance of coordinated efforts and resource pooling across Canada. While it is easy to point to post-COVID-19 pandemic vaccine hesitancy and vaccine fatigue, the more significant issues are logistical and practical barriers, including the fragmentation of the Canadian healthcare system and strategic partners. We must establish transparency and not duplicate resources. A 2020 report found that across the survey population of 2697 Canadians, only 29% of respondents had received at least one dose of the HPV vaccine [48]. Moreover, CPAC commissioned the Urban Public Health Network to coordinate a quality improvement project with Canada’s school-based HPV immunization programs. But to date, and to the best of our knowledge, the final report, as well as actional items from phase 2 of this project (which received funding of CAD two million from the CPAC), is not available in the public domain [49,50]. Taken together, these factors suggest we need to increase transparency and inter-disciplinary collaborations, e.g., those working in behavioral and implementation sciences, dentistry, infectious diseases, oncology, pharmacy, public health, sexology, and others across Canada in order to (a) get people talking about HPV vaccination, (b) educate individuals on this crucial cancer prevention vaccine, and (c) increase HPV vaccine acceptability Canada-wide.

Another recommendation is for the provincial and national dissemination of free, downloadable, and easy-to-understand educational materials from credible organizations such as HPVglobalaction.org, which offers pamphlets and posters available in 19 different languages that were developed by scientists, sexual health educators, and patients and are continuously updated and refined in accordance with the latest scientific advancements. Also, coordinated efforts like the HPV Vaccine Task Force in Ontario (a project under Immunize Canada and the Canadian Public Health Association) are excellent and could be expanded to include all of Canada, as the issues discussed apply to the entire country.

Moreover, from a practical side, many students missed their scheduled doses of the HPV vaccine due to school closures and disrupted healthcare services. Ensuring that nurses or healthcare providers visit schools regularly to administer vaccines is crucial in reaching all students. Furthermore, communication with parents needs to be clear, straightforward, and accessible. Consent forms and informational materials should be designed to be easily understood, ideally online and not on paper, removing any potential obstacles that might prevent them from accepting their children’s HPV vaccination.

The shift to a one-dose recommendation will (hopefully) have a profound positive impact on uptake. Nonetheless, Canada’s lack of national and provincial registries and issues with sharing data (as Canadians move), coupled with the need for catch-up clinics for those who missed dose(s) during the pandemic or for other reasons, is critical. There is a lack of resources and budget constraints that do not necessarily meet the needs of each individual province and Canada-wide to improve HPV vaccine uptake. As such, funding presents a key challenge to make changes to increase HPV vaccine acceptability.

The implementation of HPV DNA HCP-administered, as well as clinic- or home-based self-collection testing, can significantly increase the accessibility and convenience of screening, particularly for those who are most vulnerable. It is imperative to develop and disseminate standardized guidelines for HPV screening across all provinces and territories, ensuring a uniform approach that maximizes the effectiveness of these efforts. This includes the sharing of resources across the country. A 2020 CPAC report found that few individuals had difficulty accessing screening, with only a minority indicating barriers, such as hard-to-reach clinic locations (10%), limited hours of operation (7%), or difficulties making themselves understood by their healthcare providers (9%) [48]. The results from a national study survey of a representative sample of 3724 screening-eligible Canadians (with 1853 under-screened individuals) from diverse backgrounds aimed to understand Canadian women’s knowledge, attitudes, perceptions, and preferences regarding the forthcoming changes from primary Pap to primary HPV testing [51,52,53,54] and found that personal barriers were associated with a lower likelihood to get an HPV test. Confidence in HPV-based screening and experiencing autonomy in cervical screening (e.g., being more comfortable and in control of one’s body) was associated with higher intentions to participate in HPV-based screening for those adequately screened and both under-screened and adequately screened Canadians. This suggests that attitudinal factors (e.g., beliefs and perceived barriers) are more consistent predictors of HPV screening acceptability rather than socio-demographics or knowledge, which had mixed results. It is important that screening-eligible individuals are convinced that it is evidence-based to start screening at an increased age and with longer screening intervals compared with cytology. Messages that empower through autonomy and confidence, e.g., “cervical screening is in your hands”, “It is simple, easy, safe and can be done the comfort of your home”, and “You got this”, are encouraged. This would also include meeting women “where they are at”, understanding their experiences, and offering evidence-based solutions.

Canada’s shift to HPV testing as the primary method for cervical screening requires clarity, consistency, and collaboration in the communication of messages and implementation of this new test. Similar to the HPV vaccination task force, an inclusive, nationwide task force for HPV screening is recommended. Moreover, as home-based self-sampling is appealing to the vast majority of screening-eligible Canadians, including under-screened individuals, it should be available as a choice for all screening-eligible Canadians and not solely under-screened individuals. As such, an opt-out strategy, with the option for those who prefer provider-collected or those who, for a variety of reasons, cannot self-sample, is recommended. It is also important to note that an estimated 15% of the world’s population has a physical disability [55]. A 2022 systematic found that it is harder for disabled individuals to attend or undergo cervical screening, with barriers including false assumptions that disabled persons are not sexually active or having had a previous negative experience related to their disability [56,57]. This is a particular group worthy of attention that must not be forgotten when we think of cervical screening. Some possible issues may include getting into position or concerns about performing it correctly or harming oneself. We must include those with physical disabilities in our research studies in order to address these inequities and knowledge, attitudes, and beliefs (KABs) using reliable and validated questionnaires [58,59]. Other notable groups that have been identified but still require the development of partnerships include, but are not limited to, Indigenous communities as well as the 2SLGBTQI+ communities. Resources that incorporate the unique needs of these groups, e.g., pamphlets and posters in their native languages, with simple, understandable terminology that is inclusive and representative, are recommended.

To address misinformation, it is crucial to deliver consistent, evidence-based, and digestible public health communications. Provincial and national healthcare systems must not only identify and counteract misleading information but also provide accurate and reliable content. Highlighting the experiences of patients and families affected by cervical cancer can be particularly impactful, as personal stories often have the power to shift individual perspectives beyond mere data.

Achieving the ambitious goal of eliminating cervical cancer requires a concerted and coordinated effort across the country. This involves open communication, mobilizing efforts, and pooling resources effectively across diverse stakeholders to ensure that best practices are implemented consistently nationwide. Partnerships and collaborations between federal (e.g., the Public Health Agency of Canada), provincial, and territorial health authorities, non-governmental organizations, and community groups, including, but not limited to, the Canadian Association of Psycho-Oncology, the Canadian Cancer Society, the CPAC, the CPHA, HPV Global Action, Immunize Canada, the NACI, Young Adult Cancer Canada, and many others, can further strengthen these efforts. By sharing resources, knowledge, and expertise, we can disseminate and translate knowledge as well as implement effective programs that support the widespread adoption of HPV vaccination and screening and, ultimately, evoke a unified approach to cervical cancer prevention. This approach will also help prevent isolated efforts, un-sustained initiatives, and the inefficient use of resources.

Leveraging digital health technologies and data-sharing platforms can enhance the efficiency and reach of vaccination and screening programs, ensuring that they are accessible to all Canadians. Presently, Canada does not have a national immunization registry. In total, 11 provinces and territories in Canada, representing 85%, have electronic systems for capturing individual-level immunization data, with most (8/11) using Panorama. Each province/territory varies in terms of vaccines captured, provider delivery models, registry access, and linkages to other systems (e.g., while all registries capture vaccines administered by public health services, only some link to electronic medical records and other health systems). Despite progress in improving immunization data collection through legislation and system linkages, further advancements in immunization registries are needed to enhance system functionality, which will directly allow for comprehensive and up-to-date vaccine coverage data in order to detect differences in HPV vaccination coverage rates. This would, thereby, help individuals and/or groups that are under-immunized.

Cervical cancer is now the fastest-increasing cancer (+3.7%/year since 2015) in Canada as of 2023, marking the first significant increase in cervical cancer incidence since 1984 [60]. This is concerning to many, including the CEO of CPAC, who stated “If we are not to change our approach, that goal of elimination by 2040 could potentially be at risk”.

Delivering HPV vaccination in school-based programs is a strength across Canada. However, consent forms present a large barrier to vaccinating those eligible in schools against HPV. This is further impacted by the urgency for a centralized vaccine database, which can be seen in the differences in reporting across the country. Moreover, there is a need to involve other HCPs in the promotion of HPV vaccination, from pharmacists to dentists to psychologists to oncologists to community health workers in rural and remote areas. Since healthcare professionals have limited time, expanding outreach efforts is a crucial strategy to increase HPV vaccine uptake. Lastly, opt-in vs. opt-out policies must be considered. Simplifying consent forms will have a profound impact on uptake in Canada.

In conclusion, while there are clear targets for the next decade to move Canada toward the elimination of cervical cancer, we must have clear and specific actions including, but not limited to, Canada-wide, inclusive taskforces or gatherings on HPV vaccination and cervical screening; the dissemination of accessible, inclusive, and culturally-sensitive educational materials that speak to all Canadians; the sharing of research and coverage; framing HPV immunization as a cancer prevention vaccine; and integrating the perspectives and preferences of screening-eligible Canadians as we transition toward HPV DNA testing [51,52,53,54]. Currently, it is not necessary to survey Canadians as we already have gathered a wide variety of opinions about their KABs [51,52,53,54]. The strategy is to work together on actionable deliverables for this shift to prevent confusion and backlash and ensure that a screening-eligible individual in PEI receives the same information and recommendations as a screening-eligible individual in Manitoba, Yukon, or anywhere else in Canada. 

We must all advocate and use our voices to prevent and eliminate cervical cancer, as it is a global problem that requires global solutions. This idea is at the core of Dr. Linda Eckert’s work and book *Enough: Because We Can Stop Cervical Cancer*, which eloquently explains that cervical cancer is everybody’s issue [61]. Through collaboration and communication across all levels of government and society, we can create a future where cervical cancer is a thing of the past and Canada is a leader in cervical cancer elimination.

## Figures and Tables

**Table 1:** Most up-to-date HPV vaccination coverage rates by province.

Province	Years	School Grade When Immunization Is Given	Total (%)	Female (%)	Male (%)	Reference	Additional Remarks
**Alberta ^1^**	2021–2022	Grade 6	0.5	61.6	60.1	[19]	These coverage rates represent the proportion of 13-year-olds in the 2022–2023 school year who completed a two-dose HPV vaccine schedule.
**British Columbia ^2^**	2022–2023	Grade 6	58.85	60.0	57.7	[20]	These coverage rates represent the proportion of students in grade 6, in the 2022–2023 school year (ending 30 June 2023), that completed a three-dose HPV vaccine schedule.
**Manitoba**	2022–2023	Grade 6	53.35	54.4	52.3	[21]	Currently undergoing a review of the immunization coverage data [22].
**New Brunswick**	2022–2023	Grade 7	69.6	66.3	73	[23]	These coverage rates represent the proportion of students in grade 7, in the 2022–2023 school year, that completed a two-dose HPV vaccine schedule.
**Newfoundland**	2020–2021	Grade 6	76.1			[24]	The pandemic affected the availability of resources, and efforts are underway to acquire more recent years’ uptake rates. Currently, there is no timeline for when the rates may be available [24]
**Northwest Territories**	2015–2016	Grades 4–6		57.1		[25]	Recent data were not able to be found by the author.
**Nunavut**	2015–2019		~57			[25]	Recent data were not able to be found by the author. Rates of 61.5% for 3 doses in 2014–2015 were reported [10].
**Nova Scotia**	2021–2022	Grade 7	81.5	80.5	82.4	[26]	These coverage rates represent the proportion of students in grade 7, in the 2021–2022 school year, that completed a two-dose HPV vaccine schedule-
**Ontario ^3^**	2022–2023	Grade 7	47.9			[27]	These coverage rates represent the proportion of 12-year-old students, in the 2022–2023 school year, that completed a two-dose HPV vaccine schedule.
**Prince Edward Island**	2022–2023	Grade 6	81.4	83.7	80.2		These coverage rates represent the proportion of Grade 6 students, in the 2019–2020 school year, that completed a two-dose HPV vaccine schedule.
**Québec ^4^**	2022–2023	Grade 4	81.0	83.0	80.0	[28]	These coverage rates represent the proportion of Grade 4 students, in the 2022–2023 school year, that completed a two-dose HPV vaccine schedule.
**Saskatchewan ^5^**	2022–2023	Grade 6		62.2	61.8	[29]	These coverage rates represent the proportion of 13-year-old students, in the 2022–2023 school year, that completed a two-dose HPV vaccine schedule.
**Yukon**	2012	Grade 6	~50%				

^1^ The rates for one dose in the 2022–2023 school year was 69.2% for 13-year-old females and 68.9% for 13-year-old males. The proportion of 17-year-olds in the 2022–2023 school year who completed one dose of the HPV vaccine was 61.6% for females and 77.3% for males. The rate for two doses of the HPV vaccine for 17-year-olds in 2022–2023 for females was 74.0, and it was 74.2% for males. ^2^ The rates for one dose for Grade 6 students in the 2022–2023 school year were 15.5% for females and 15.9% for males. The proportion of Grade 9 students in the 2022–2023 school year who completed one dose was 11.1% for females and 12.1% for males. The proportion of students in Grade 9 in the 2022–2023 school year who completed a three-dose HPV vaccine schedule was 73.7% for females and 70.8% for males. ^3^ The proportion of 12-year-olds in the 2022–2023 school year who completed a three-dose HPV schedule was 0.1%. The proportion of 12-year-olds in the 2022–2023 school year who completed a one-dose HPV schedule was 17.8%. The proportion of 13-year-olds in the 2021–2022 school year who completed two or three doses was 34.8%. The up-to-date immunization coverage estimates reflect the proportion of students who completed the series and received all recommended doses for their age by August 31st of the respective school year or catch-up period (i.e., 1, 2, or 3 years following the corresponding school year), which for 2021–2022, was 16.6%. The proportions of 13-, 14-, 15-, 16-, and 17-year-olds in the 2022–2023 school year who completed either a two- or three-dose HPV schedule were 51.6%, 40.7%, 47.4%, 67.9%, and 68.5%, respectively. ^4^ The proportion of Grade 9 students in the 2022–2023 school year who completed a two-dose HPV schedule was 83% overall, with 85% for females and 82% for males. The proportion of 12-year-olds in the 2022–2023 school year who completed a one-dose HPV schedule was 17.8%. The proportion of 13-year-olds in the 2021–2022 school year who completed two or three doses was 34.8%. ^5^ The proportion of 13-year-old students in the 2022–2023 school year who completed a one-dose HPV schedule was 74.8% for females and 74.7% for males. The proportion of 15-year-olds in the 2022–2023 school year who completed a one-dose HPV schedule was 82.7% for females and 81.5% for males, and for a two-dose schedule, it was 74.4% for females and 71.8% for males. The proportion of 17-year-olds in the 2022–2023 school year who completed a one-dose HPV schedule was 81.7% for females and 81% for males, and for a two-dose schedule, it was 76.3% for females and 75.1% for males. For 13-, 15-, and 17-year-olds, the three-dose rates were between 0.1% and 1.1%.

**Table 2:** Most up-to-date statuses of cervical screening by province.

Province	Current Status	Additional Comments	Reference
**Northwest Territories**	To the best of our knowledge, no current HPV primary screening activities.		Recent information was not able to be found by the author.
**Alberta**	Planning for implementation and have plans to implement self-sampling to facilitate HPV primary screening implementation.	For HPV self-sampling, three targeted populations have been identified for a pilot project: those who identify as Indigenous, rural remote, and newcomers. They plan to roll out 5000 test kits between May 2024 and 2026. The HPV self-sampling pilot is a partnership with Alberta Health Services and is funded by Alberta Health.	[39]
**British Columbia**	Jurisdiction-wide implementation of HPV testing and using self-sampling to facilitate HPV primary screening implementation.	There was a pilot program launched in December 2021 including people who were overdue for a Pap test in September 2022. Jurisdiction-wide implementation began on 29 January 2024, when all eligible women will be able to order an HPV self-sampling test kit at home.	[40]
**Manitoba**	Piloting.		[39]
**New Brunswick**	Planning for implementation and have plans to implement self-sampling to facilitate HPV primary screening implementation.	An advisory group is being established, and work on the transition is expected to begin in the fall of 2023.	[41]
**Newfoundland**	Piloting, and have plans to implement self-sampling to facilitate HPV primary screening implementation.	Plans to submit a proposal in autumn 2023.	[42]
**Nova Scotia**	Planning for implementation.	The team currently manages three cancer screening programs for lung, colorectal, and cervical cancers. They are still in the planning stages. Nova Scotia Health Cancer Care Program declined to analyze provincial attitudes and preferences data made available to them by Nova Scotia screening eligible individuals to support the transition to HPV testing.	[43]
**Nunavut**	To the best of our knowledge, no current HPV primary screening activities.		Recent information could not be found by the author.
**Ontario**	Planning for implementation.		Recent information could not be found by the author.
**Prince Edward Island**	Province-wide HPV DNA testing implemented and have plans to implement self-sampling to facilitate HPV primary screening implementation.	Since May 2024, they have fully transitioned to the HPV primary screening method. The self-sampling HPV screening method is currently being validated by their labs, and a pilot might move forward by 2025.	[44]
**Québec**	Partially implemented	A deployment schedule for the first-line HPV test has been developed. They are prioritizing regions that are experiencing delays in reading Pap tests. Work is ongoing within laboratory clusters to adapt to the arrival of liquid cytology. HPV testing has started to roll out in three regions: Bas-Saint-Laurent, Gaspésie, and Chaudière-Appalaches.	[45]
**Saskatchewan**	Piloting (and have plans to implement self-sampling to facilitate HPV primary screening implementation)	Loosely defined plans to plans to run a survey with hard-to-reach populations (*n* = 100) to understand the beliefs of eligible women. The Saskatchewan Cancer Agency declined to partner to analyze provincial attitudes and preferences data made available to them by Saskatchewan screening eligible individuals to support the transition to HPV testing.	[46]
**Yukon**	Planning for implementation.		Recent information was not able to be found by the author.

## Data Availability

Data sharing is not applicable to this article.
